# Supplementary figures and images for: Translational gene expression control in Chlamydia trachomatis
Source: PLoS One. 2022 Jan 27;17(1):e0257259. doi: 10.1371/journal.pone.0257259 (PMC8794103; doi:10.1371/journal.pone.0257259)

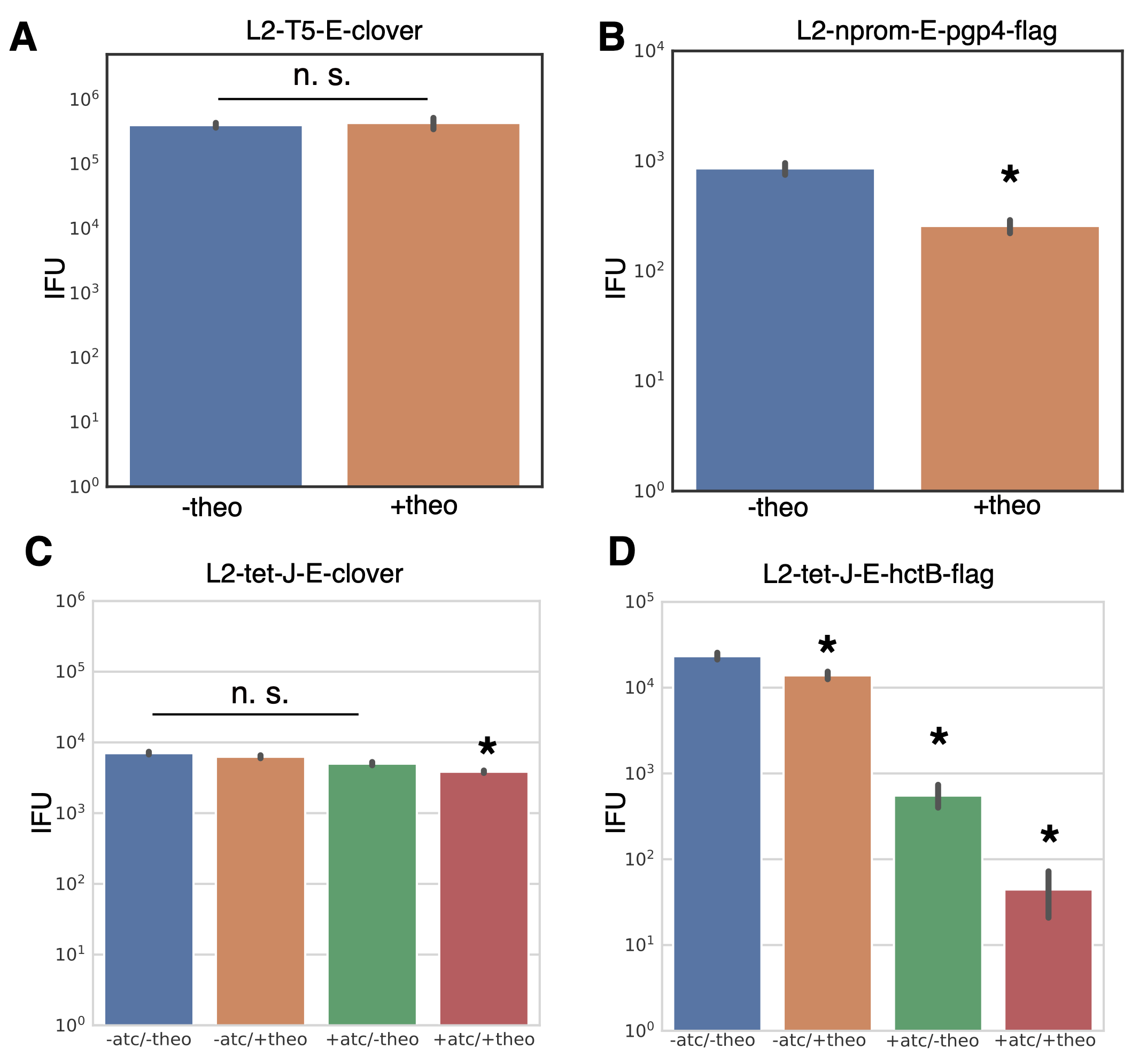

Supplement: S1 Fig — A) Cos-7 cells were infected with L2-T5-E-clover-flag and the production of infectious progeny was determined at 30 hpi after theophylline induction or vehicle only. B) Cos-7 cells were infected with L2-nprom-E-pgp4-flag and the production of infectious progeny was determined at 30 hpi after theophylline induction or vehicle only. C) Cos-7 cells were infected with L2-Tet-J-E-clover-flag and the production of infectious progeny was determined at 30 hpi after induction with 0.5 mM theophylline, 30ng/ml aTc, both aTc and theophylline or vehicle only at 16 hpi. D) Cos-7 cells were infected with L2-Tet-J-E-hctB-flag and the production of infectious progeny was determined at 30 hpi after induction with 0.5 mM theophylline, 30ng/ml aTc, both aTc and theophylline or vehicle only at 16 hpi. Asterisks denote p-values < 0.05. Error bars = SEM. (TIF) [file pone.0257259.s001.tif]

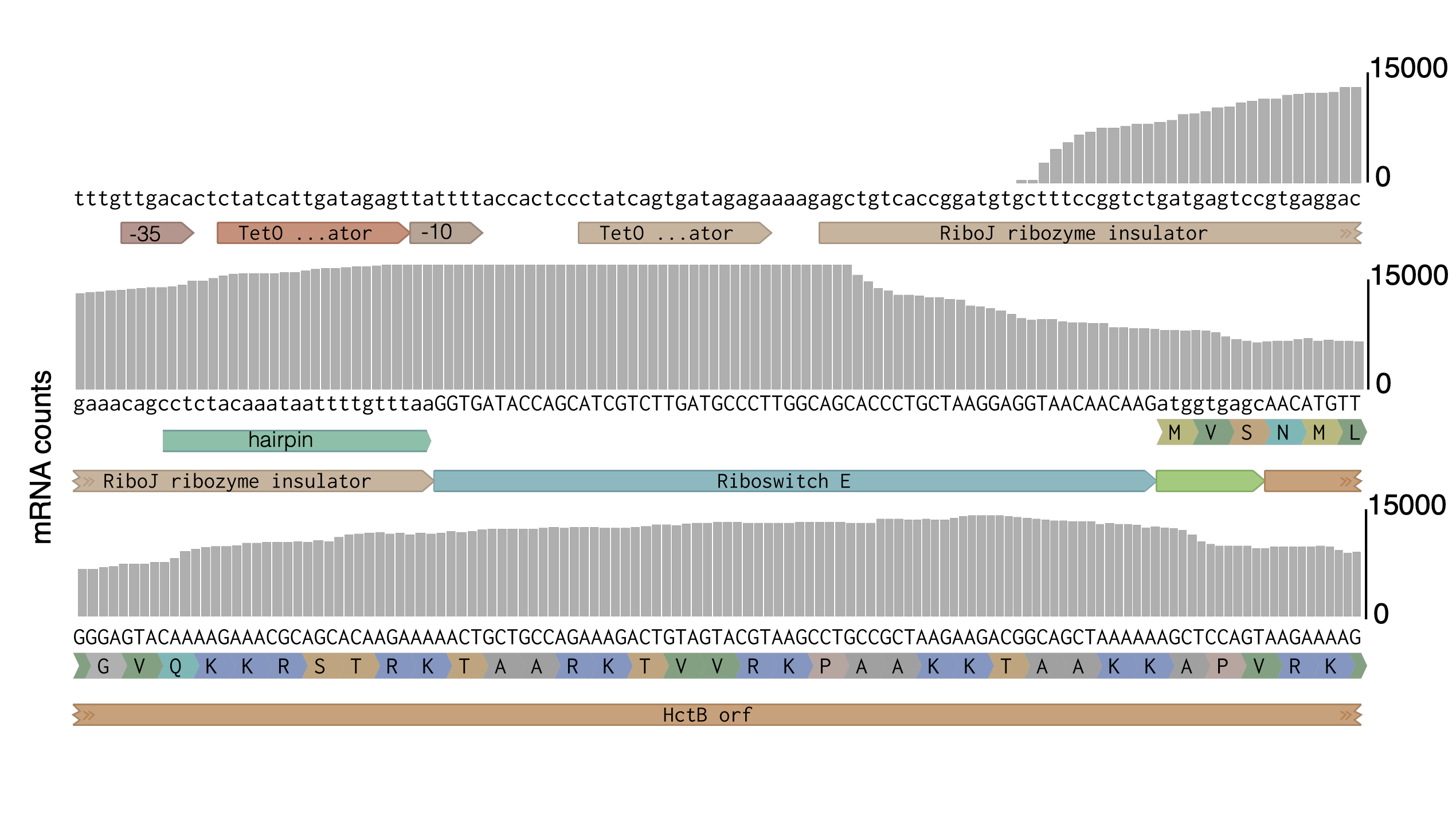

Supplement: S2 Fig — Cos-7 cells infected with L2-Tet-J-E-hctB-flag were induced with 0.5 mM theophylline and 30ng/ml aTc at 15 hpi and RNA was harvested at 24 hpi. RNA was processed for next-gen RNA-seq sequencing. Aligned reads are shown with the schematic of the Tet-J-E-hctB. (TIF) [file pone.0257259.s002.tif]

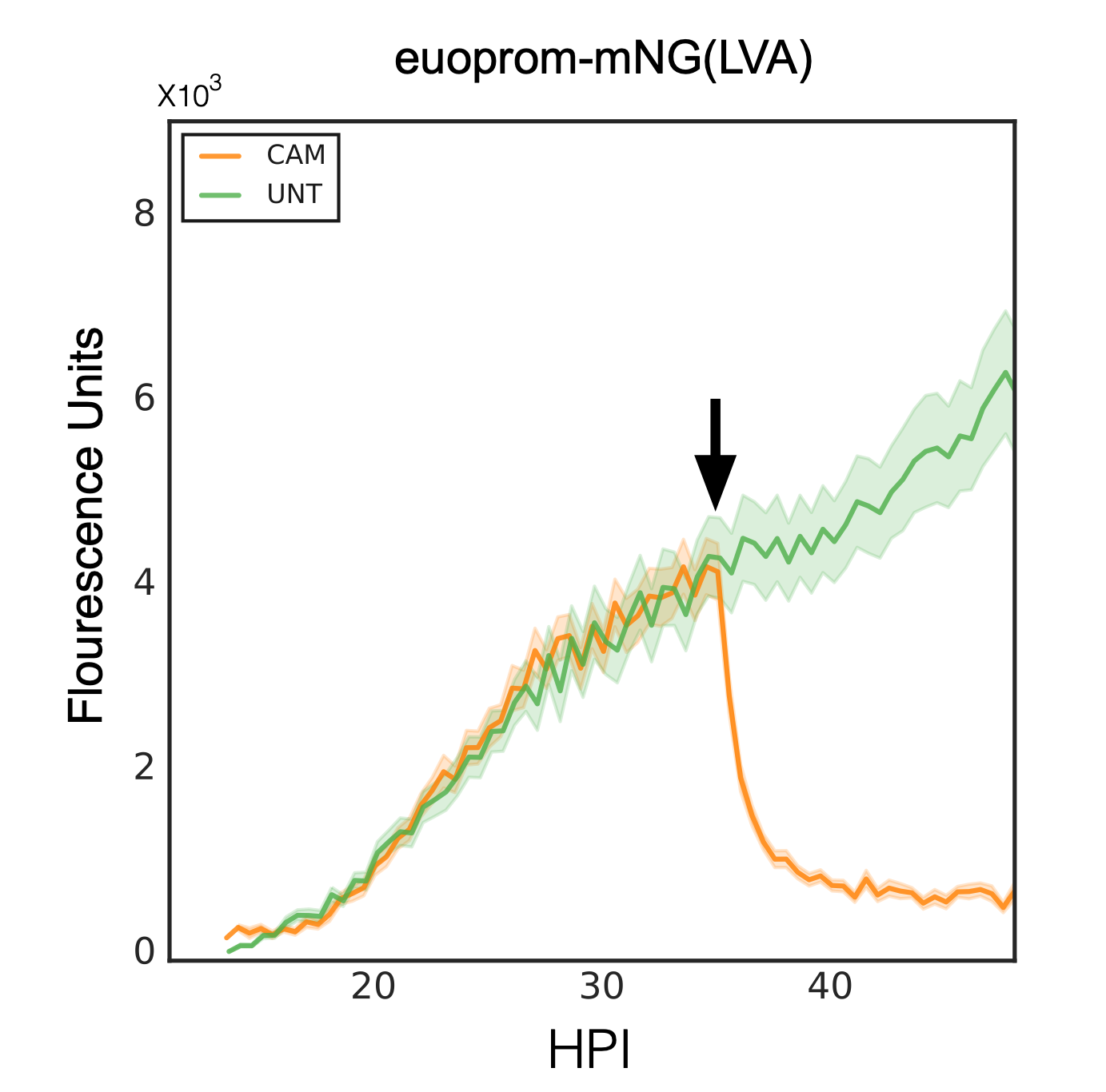

Supplement: S3 Fig — Cos-7 cell infected with L2-euoprom-ngLVA, treated with 34 μg/ml chloramphenicol (CAM) or vehicle only (1:1000 EtOH in RPMI) at 35 hpi (arrow). The infections were monitored using live cell imaging for 50 hours. Expression intensities from >50 individual inclusions were measured via automated live-cell fluorescence microscopy and the mean intensities are shown. Cloud represents SEM. Y-axes are denoted in scientific notation. Chloramphenicol treated sample showed a decrease of half max intensity 30 mins after treatment. (TIFF) [file pone.0257259.s003.tiff]
