# Supplementary material for: Translational gene expression control in Chlamydia trachomatis
Source: PLoS One. 2022 Jan 27;17(1):e0257259. doi: 10.1371/journal.pone.0257259 (PMC8794103; doi:10.1371/journal.pone.0257259)

Probed for Flag Tag:
Samples: 1) E-Clover: -Theo
2) E-Clover: +Theo

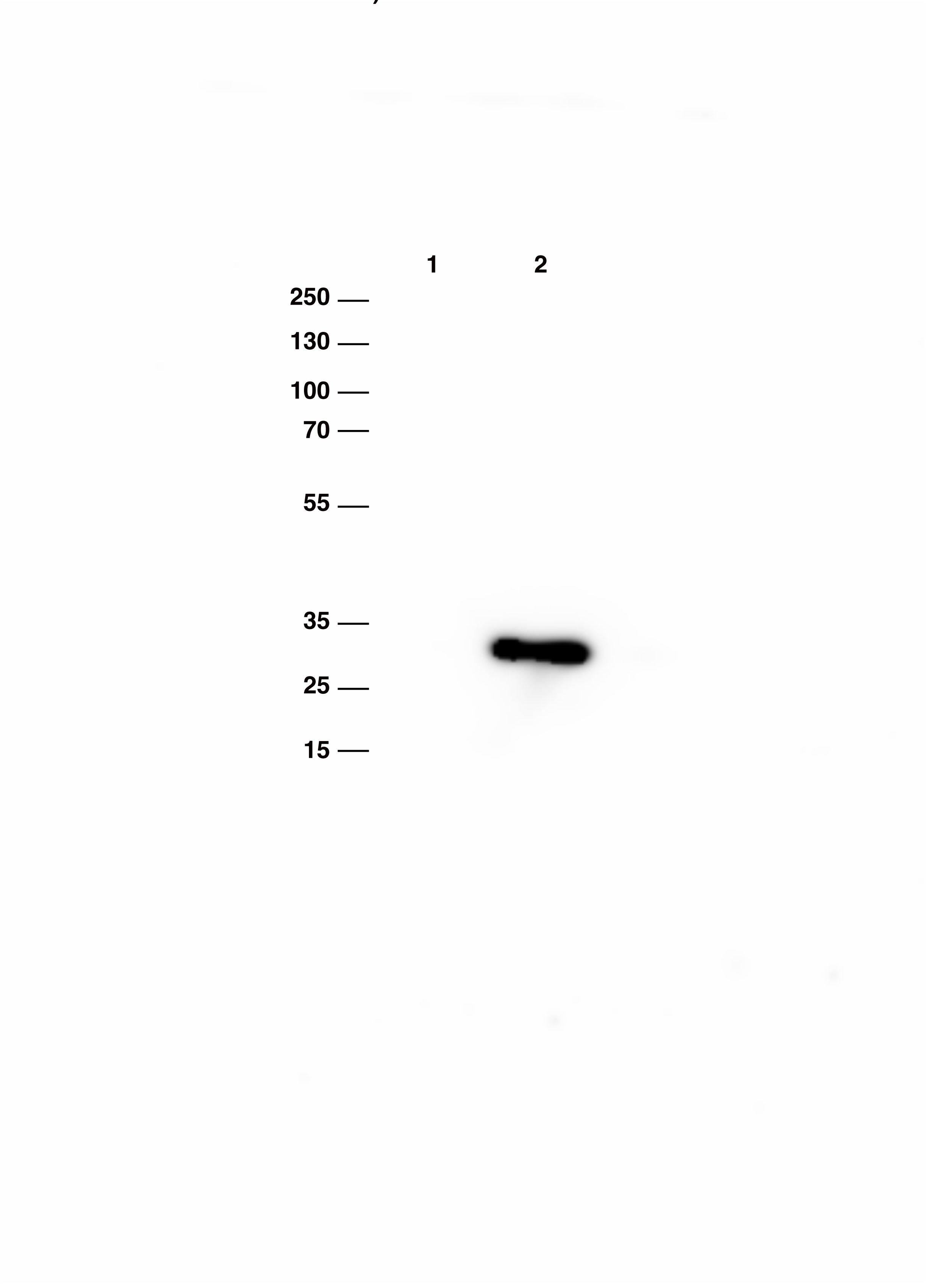

Probed for Flag Tag:
Samples: 1) E-Pgp4: -Theo
2) E-Pgp4: +Theo

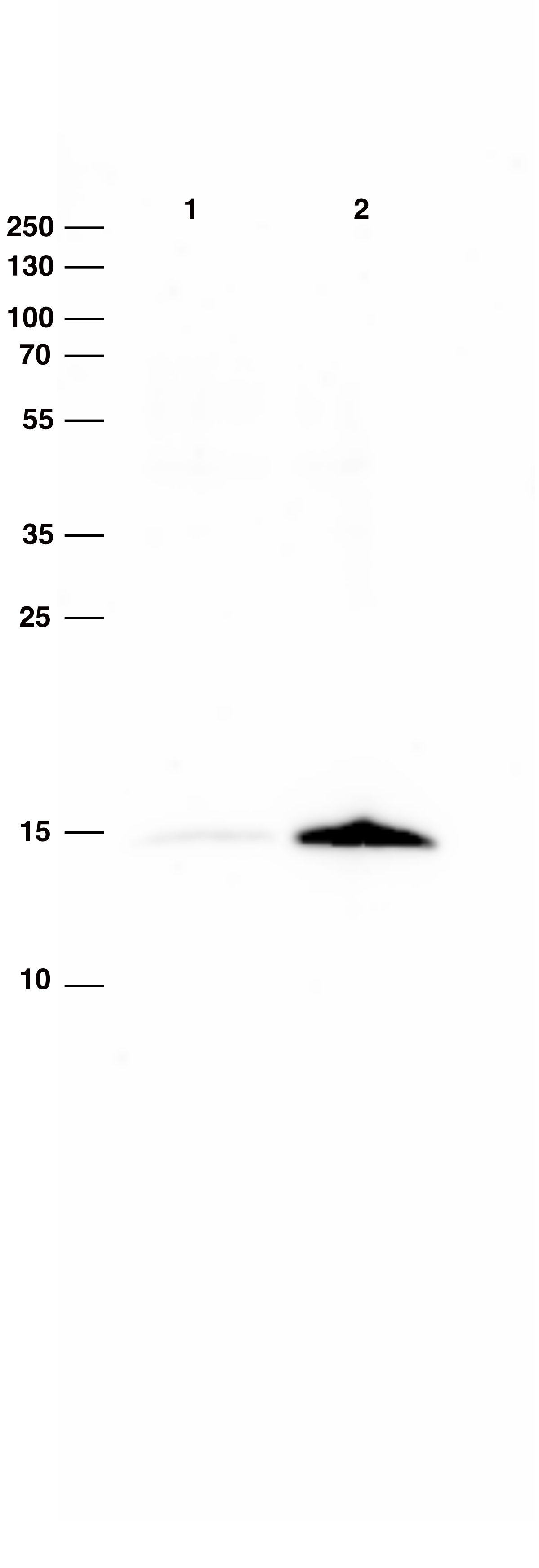

Probed for Flag-Tag:

Samples: 1) Tet-E-Clover: -aTc/-Theo

2) Tet-E-Clover: -aTc/+Theo

3) Tet-E-Clover: +aTc/-Theo

4) Tet-E-Clover: +aTc/-Theo

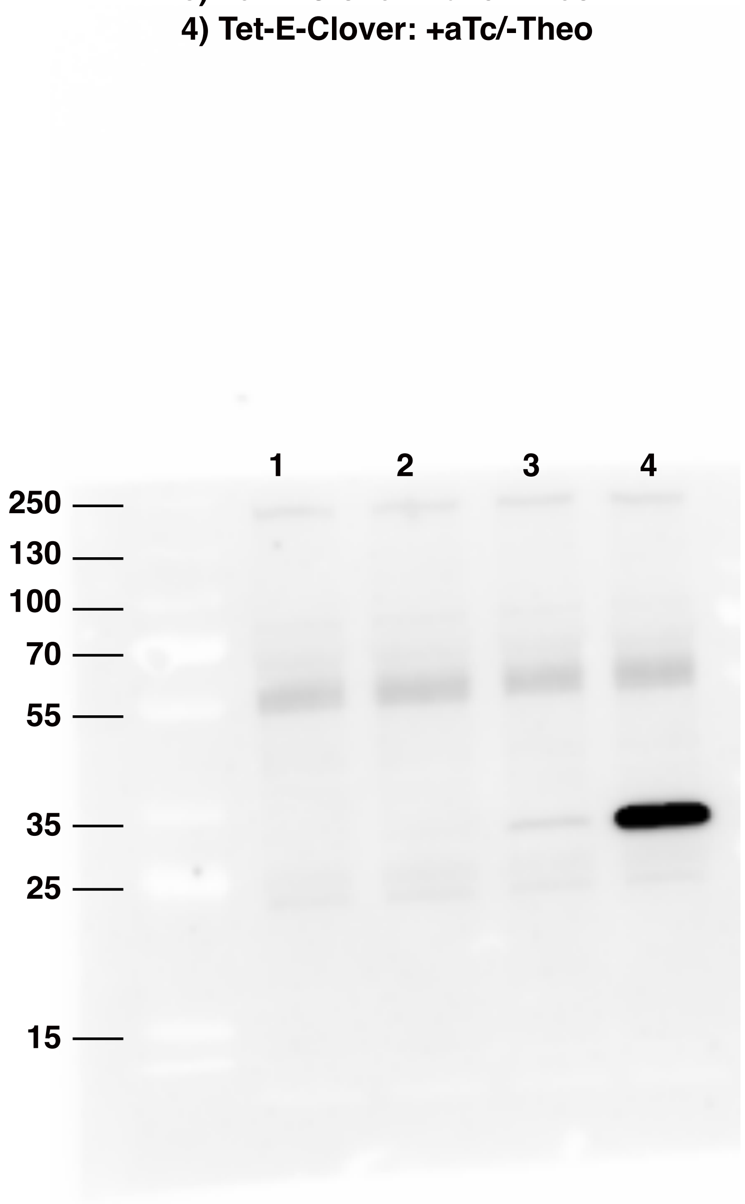

Probed for Flag-Tag:

Samples: 1) Tet-E-HctB: -aTc/-Theo
2) Tet-E-HctB: -aTc/+Theo
3) Tet-E- HctB: +aTc/-Theo
4) Tet-E-HctB: +aTc/-Theo

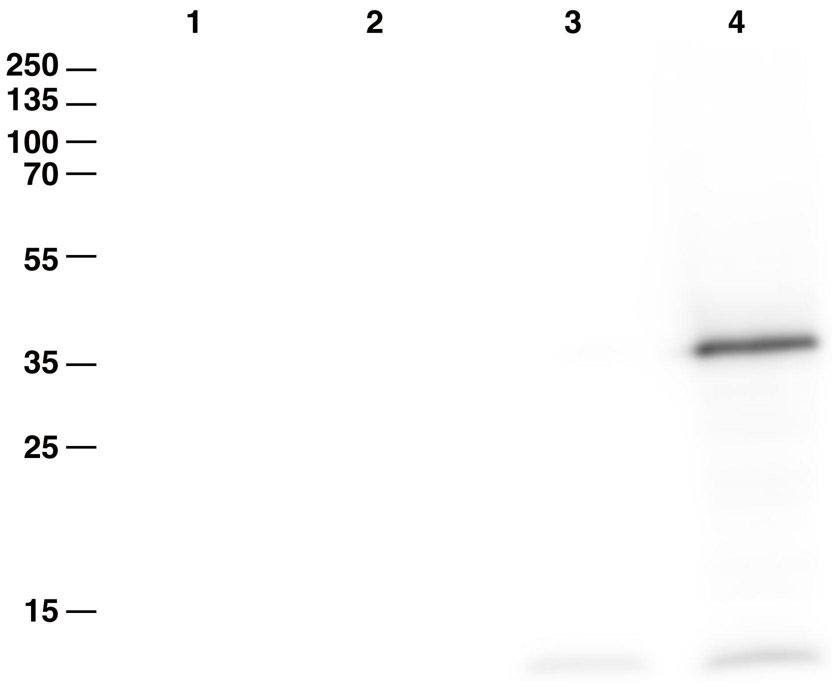

Probed for Beta Tubulin I:

Samples: 1) Tet-E-Clover: -aTc/-Theo
2) Tet-E-Clover: -aTc/+Theo
3) Tet-E-Clover: +aTc/-Theo
4) Tet-E-Clover: +aTc/-Theo
5) Blank lane
6) E-Clover: -Theo
7) E-Clover: +Theo

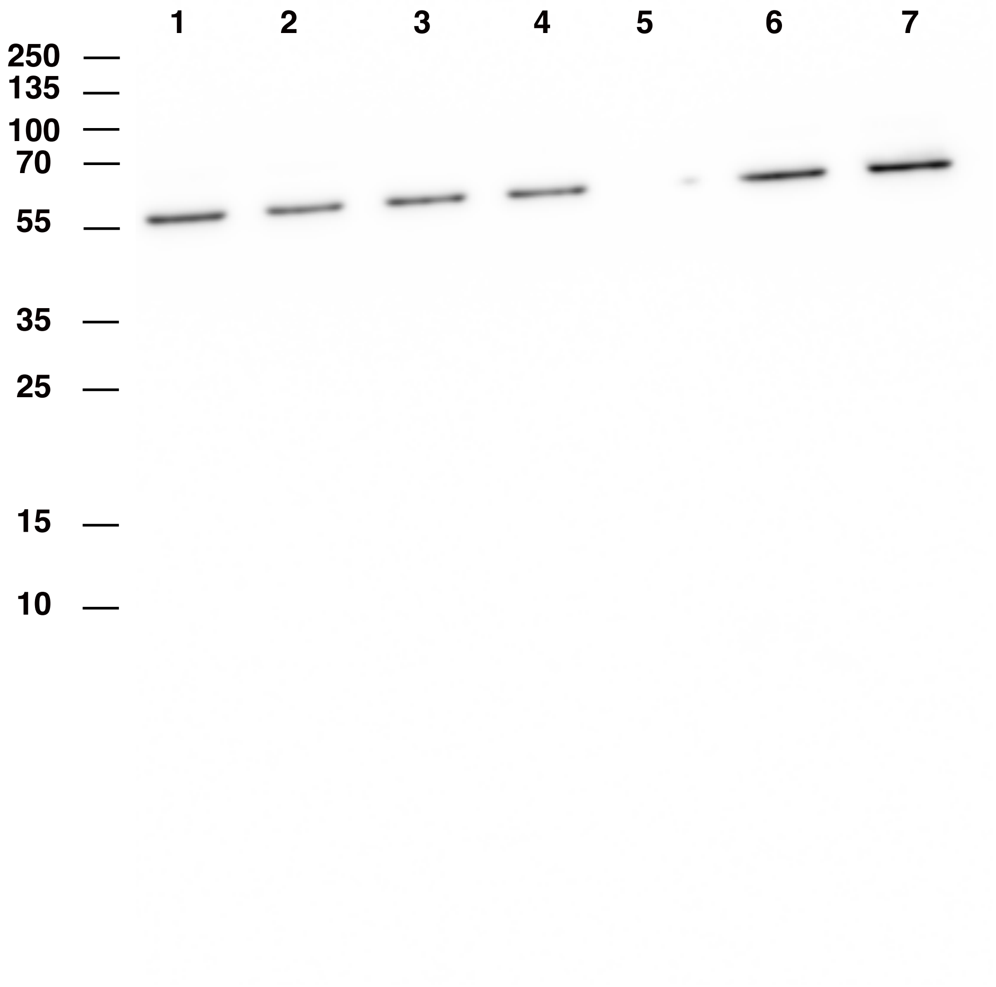

Probed for Beta Tubulin I:

Samples: 1) Tet-E-HctB: -aTc/-Theo

2) Tet-E-HctB: -aTc/+Theo

3) Tet-E- HctB: +aTc/-Theo

4) Tet-E-HctB: +aTc/-Theo

5) E-Pgp4: -Theo

6) E-Pgp4: +Theo

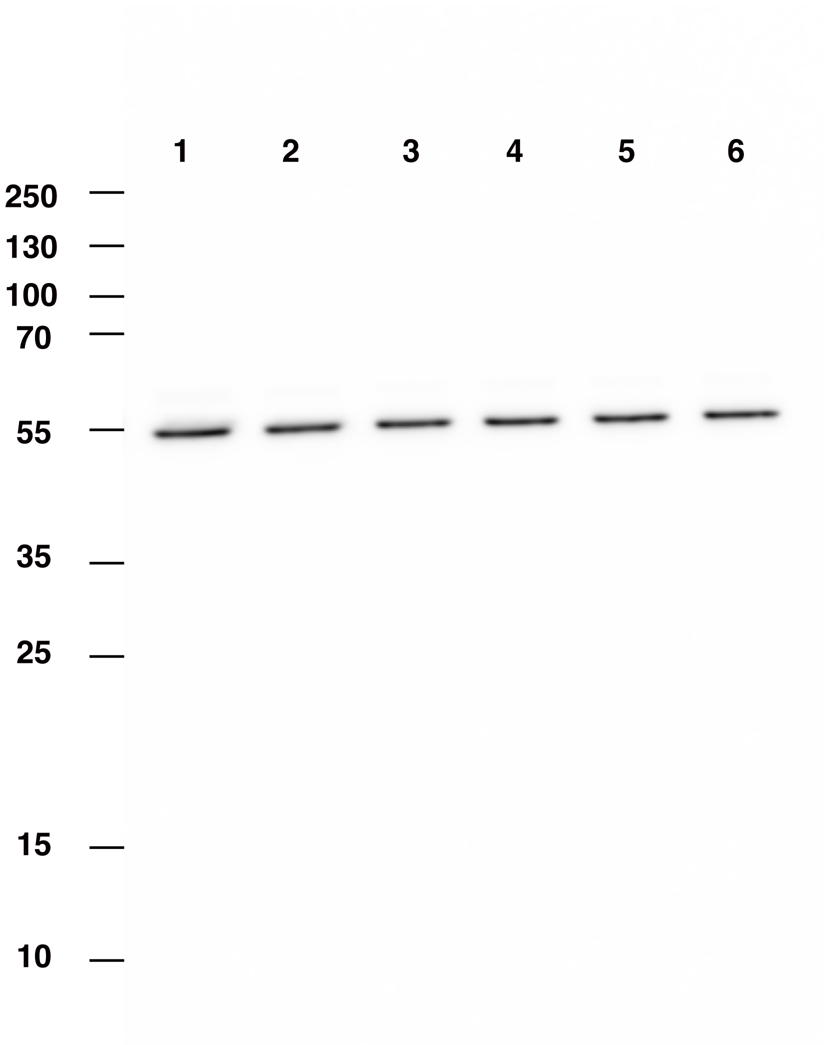

Supplement: S4 Fig — (PDF) [file pone.0257259.s004.pdf]
