# Supplementary material for: Translational gene expression control in Chlamydia trachomatis
Source: PLoS One. 2022 Jan 27;17(1):e0257259. doi: 10.1371/journal.pone.0257259 (PMC8794103; doi:10.1371/journal.pone.0257259)
Supplement: S1 Table — (PDF) [file pone.0257259.s005.pdf]

Sequence	Primer name	Template
**T5-E-Clover-3xFlag**		
ctataggtaccggtgataccagcatcgtcttgatgcccttggcagcaccctgctaaggaggtaacaacaagatggtgagcaagggcgaggagctgttcaccgg<br>ggtggtgcccatcctggtcgagctggacggcgacgtaaacggccacaagttcagcgtccgcggcgagggcgagggcgatgccaccaacggcaagctgaccctg<br>aagttcatctgcaccaccggcaagctgcccgtgccctggcccaccctcgtgaccaccttcggctacggcgtggcctgcttcagccgctaccccgaccacatga<br>agcagcacgacttcttcaagtccgccatgcccgaaggctacgtccaggagcgcaccatctctttcaaggacgacggtacctacaagacccgcgccgaggtgaa<br>gttcgagggcgacaccctggtgaaccgcatcgagctgaagggcatcgacttcaaggaggacggcaacatcctggggcacaagctggagtacaacttcaacagc<br>cacaacgtctatatcacggccgacaagcagaagaacggcatcaaggctaacttcaagatccgccacaacgttgaggacggcagcgtgcagctcgccgaccact<br>accagcagaacacccccatcggcgacggccccgtgctgctgcccgacaaccactacctgagccatcagtccgccctgagcaaagaccccaacgagaagcgcga<br>tcacatggtcctgctggagttcgtgaccgccgccgggattacacatggcatggacgagctgtacaagatggactacaaagaccatgacggtgattataaagat<br>catgacatcgattacaaggatgacgattaaggatgac	gBlock E-Clover-Flag	N/A
cgattaaggatgacatgtgattcgcgtagga	5' E-clover-Flag bb	p2TK2-SW2
tcaccggtacctatagtgagtcgtatctctagtaattgttatcc	3' E-Clover-Flag bb	
**Tet-riboJ-E-clover-3xFlag**		
tgtggataaccgtattacatttaagacccactttcacatttaagttgtttttctaatccgcatatgatcaattcaaggccgaataagaaggctggctctgcac<br>cttggtgatcaaataattcgatagcttgtcgtaataatggcggcatactatcagtagtaggtgtttccctttcttctttagcgacttgatgctcttgatcttc<br>caatacgcaacctaaagtaaaatgccccacagcgctgagtgcatataatgcattctctaatgaaaaaccttgttggcataaaaaggctaattgattttcgaga<br>gtttcatactgtttttctgtaggccgtgtacctaaatgtacttttgctccatcgcgatgatttagtaaagcacatctaaaacttttagcgttattacgtaaaa<br>aatcttgccagctttccccttctaaagggcaaaagtgagtatggtgcctatctaacatctcaatggctaaggcgtcgagcaaagcccgcttattttttacatg<br>ccaatacaatgtaggctgctctacacctagcttctgggcgagtttacgggttgttaaaccttcgattccgacctcattaagcagctctaatgcgctgttaatc<br>actttacttttatctaatctagacatcattaattcctaatttttgttgacactctatcattgatagagttattttaccactccctatcagtgatagagaaaag<br>agctgtcaccggatgtgctttccggtctgatgagtccgtgaggacgaaacagcctctacaaataattttgtttaaggtgataccagcatcgtcttgatgccct<br>tggcagcaccctgctaaggaggtaacaacaagatggtgagcaagggcgagga	gBlock Tet-riboJ-E	N/A
atggtgagcaagggcgaggagctgttcacc	5' tet-riboJ-E bb	T5-E-Clover-3xFlag
atgtaatacggttatccacagaatcaggggataacg	3' tet-riboJ-E bb	
**T5-E-hctB-3xFlag**		
gatggtgagcaacatgttgggagtacaaaagaaacgcagc	5' E-HctBi	L2 genomic
tgtagtccattctagcgactaatttcattaattgttgacgccagctgtgagc	3' E-HctBi	
agtcgctagaatggactacaaagaccatgacggtgattataaagatcatgacatcg	5' E-HctB bb	T5-E-Clover-3xFlag
ccaacatgttgctcaccatcttgttgttacctccttagcagggtgc	3' E-HctB bb	
**Tet-riboJ-E-hctB-3xFlag**		
gatggtgagcaacatgttgggagtacaaaagaaacgcagcac	5' tet-riboJ-HctBi	L2 genomic
tgtagtccattctagcgactaatttcattaattgttgacgccagctgtgagc	3' tet-riboJ-HctBi	
agtcgctagaatggactacaaagaccatgacggtgattataaagatcatgacatcg	5' tet-riboJ-HctB bb	Tet-riboJ-E-Clover-3xFlag
ccaacatgttgctcaccatcttgttgttacctccttagcagggtgc	3' tet-riboJ-HctB bb	
**nprom-E-pgp4-3xFlag**		
tatttagagaaaacagttcgggtgataccagcatcgtcttgatgcccttggcagcaccctgctaaggaggtaacaacaagatgcaaaataaaagaaaagtgag<br>ggacgattttattaaaattgttaaagatgtgaaaaaagatttccccgaattagacctaaaaatacgagtaaacaaggaaaaagtaactttcttaaattctccc<br>ttagaactctaccataaaagtgtctcactaattctaggactgcttcaacaaatagaaaactctttaggattattcccagactctcctgttcttgaaaaattag<br>aggataacagtttaaagctaaaaaaggctttgattatgcttatcttgtctagaaaagacatgttttccaaggctgaaatggactacaaagaccatgacggtga<br>ttataaagatcatgacatcgattacaaggatgacgattaaacaacttact	gBlock E-pgp4-flag	N/A
cgaactgttttctctaaatataaaacctataagaaaaatccaataaaaattgtttaagc	5' E-pgp4-flag bb	p2TK2-SW2
tgacgattaaacaacttactctaacgttggagttgatttgcacaccttag	3' E-pgp4-flag bb	
**T5-E-ngLVA-3xFlag, Tet-J-E-ngLVA-3xFlag**		
atggtgagcaaaggcgaagaagataacatggcgagcctgccggcgacccatgaactgcatatttttggcagcattaacggcgtggattttgatatggtgggcc<br>agggcaccggcaacccgaacgatggctatgaagaactgaacctgaaaagcaccaaaggcgatctgcagtttagcccgtggattctggtgccgcatattggcta<br>tggctttcatcagtatctgccgtatccggatggcatgagcccgtttcaggcggcgatggtggatggcagcggctatcaggtgcatcgcaccatgcagtttgaa<br>gatggcgcgagcctgaccgtgaactatcgctatacctatgaaggcagccatattaaaggcgaagcgcaggtgaaaggcaccggctttccggcggatggcccgg<br>tgatgaccaacagcctgaccgcggcggattggtgccgcagcaaaaaaacctatccgaacgataaaaccattattagcacctttaaatggagctataccaccgg<br>caacggcaaacgctatcgcagcaccgcgcgcaccacctatacctttgcgaaaccgatggcggcgaactatctgaaaaaccagccgatgtatgtgtttcgcaaa<br>accgaactgaaacatagcaaaaccgaactgaactttaaagaatggcagaaagcgtttaccgatgtgatgggcatggatgaactgtataaaggcctgcagcaa<br>acgacgaaaactacgctttagtagcttag	gBlock neongreenLVA	N/A
agtagcttagggatgacatgtgattcgcgtaggaaaaagaggaggg	5' ngLVA bb (T5 and Tet)	Tet-riboJ-E-Clover-3xFlag
tgctcaccatcttgttgttacctccttagcagggtgctgccaagg	3' ngLVA bb (T5 and Tet)	or T5-E-Clover-3xFlag
**euoprom-ngLVA**		
taacaacaagatggtgagcaaaggcgaagaagataacatggcgagc	5' ngLVAi	E-ngLVA-3xFlag
catgtcatccctaagctactaaagcgtagttttcgtcgtttgctgcagg	3' ngLVAi	
aaaactacgctttagtagcttaggacggatcccttgtacaatcaatttaccgattaaatagtctc	5' euo ngLVA bb	euoprom-Clover[18]
gcttacaacaagatacaggggtcgaattcggcatggtgagca	3' euo ngLVA bb	
