# Supplementary material for: Translational gene expression control in Chlamydia trachomatis
Source: PLoS One. 2022 Jan 27;17(1):e0257259. doi: 10.1371/journal.pone.0257259 (PMC8794103; doi:10.1371/journal.pone.0257259)
Supplement: Attachment — Submitted filename: Reviews comments to Fancytet.docx [file pone.0257259.s006.docx]

We have addressed the reviews comments as follows:

Both reviewers asked for a Chlamydia loading control. These concerns need to be addressed with the relevant data provided in a revised MS

We have provided a tubulin control as loading controls for the westerns. We chose to not use a chlamydial antigen as induction of both Pgp4 and HctB affect the developmental cycle of chlamydial making any chlamydial protein a poor choice for a loading control.

**Comments to the Author**

Reviewer #1: In this manuscript by Grieshaber et al., the authors describe a new method of translational control, riboswitch E, for use in Chlamydia trachomatis. The ability to control translation of chlamydial factors will enable functional characterization of regulatory factors and effectors. Overall the manuscript is well written and easy to follow, however I have some concerns related to data presentation.

**Major comments:**

Fig 1B, Fig. 3B, Fig. 4B, etc.: **A loading control** (Ctr HSP) should be included.

We have added beta tubulin control as loading controls for the westerns. We chose not to use a chlamydial antigen as induction of both Pgp4 and HctB affect the developmental cycle of chlamydial making any chlamydial protein a poor choice for a loading control.

In my opinion, Fig. 2A should be part of Fig. 1 (maybe Fig. 1D?). The bar graph should also have N.S. to indicate no significant difference following Theo treatment.

We agreed and updated the figure and text. We have added N. S. Where appropriate

The decimal seems to be missing in the legend in Fig. 2B. Should also include mM in the legend.

Fixed

Fig. 7 should have N.S. on graphs to indicate no significant difference.

This is now addressed

Scale bars are missing from many of the microscopy images.

This is now addressed

Details on the number of experimental replicates and/or technical replicates is missing. This should be included in the figure legends or materials and methods section.

This is now addressed

**Minor comments:**

In many places, Chlamydia is italicized but trachomatis is not. Both should be italicized.

This is now addressed

In some instances, the authors use Chlamydia trachomatis whereas in others they use Ctr. Consistent use of one or the other should be used.

We are now consistently using Ctr

Line 237, 454 should be 434

fixed

**Reviewer #2:** Greishaber et al. describe the development of a theophylline-inducible riboswitch E that allows for regulatable translation in Chlamydia trachomatis L2 from plasmid-encoded genes. Riboswitch-regulation was assessed with both native (pgp4) and synthetic promoters (T5-lac and tet). The tet-promoter system allowed for transcriptional control (tet operator/repressor) in addition to translational control via the riboswitch. In general, the data are convincing and regulation appears to be very tight and dose-responsive. The riboswitch approach should have significant utility for the chlamydial field as highlighted by the construction of hctB-plasmid transformants using the dual tet-riboswitch E vector to negate HctB-toxicity owing to leaky expression. In addition, the authors document that the T5-lac/tet promoters appear to show developmental-stage specific expression which has relevance for many of the chlamydial expression-vectors in use. Suggestions for improving the manuscript are listed below as major and minor criticisms.

**Major criticisms**

1) The Flag-tag western blots showing induction of the protein of interest in figures 1, 3, 4, and 6 would greatly benefit from **loading controls** to demonstrate that chlamydial proteins were present in the Flag-tag negative lanes. Inclusion of sample-matched anti-MOMP western blots (or another prominent chlamydial protein) would address this weakness.

We have added a beta tubulin control as loading controls for the westerns. We chose not to use a chlamydial antigen as induction of both Pgp4 and HctB affect the developmental cycle of chlamydial making any chlamydial protein a poor choice for a loading control.

2) The group has already reported the use of the riboswitch E in a recent publication. The study is referenced in the methods regarding how transformations were performed (ref 38, line 132), but nowhere else. It is clear that the current manuscript is an in-depth analysis of the riboswitch and it provides significant new details that merit sharing with the field. **A brief discussion of the use of the riboswitch in the prior study would further support the utility of the system** and it is recommended that the authors highlight the prior usage with dbdA, hctA, and scc1.

We have added this to the discussion

Minor criticisms

1) The RNAseq data under SRA 10220676 ([https://www.ncbi.nlm.nih.gov/sra/?term=10220676](https://urldefense.com/v3/__https://www.ncbi.nlm.nih.gov/sra/?term=10220676__;!!JYXjzlvb!xFOfLjX23jcw_XRc4BUYvcfDPYPJCB4UGZpvzGB2yXPdcVIYY7CkhTsw3spUQU8k$), accessed on 9-30-21) do not appear to match the current study. Please confirm the submission number.

Our mistake, this was the temporary accession number the correct one is PRJNA756251

The methods mention using pen G or spec as appropriate for the plasmids (lines 133-135). All of the plasmids reported appear to be p2TK2-SW2 based (ref 20), which uses the bla marker. Please indicate which vector required spec-selection.

We have updated the material and methods to reflect that we replaced bla with spec in this construct

3) Please state in the methods the vehicle used to dissolve theophylline.

Theophylline was dissolved straight into RPMI media. We have added this to the materials and methods

4) Line 265 – “two” should be too

Fixed

5) The methods state that glycogen staining was performed at 40 hpi (line 197), while the results indicate 36 hpi (328). Please clarify.

This was done at 36 hours and has been updated in the materials and methods

6) When discussing the toxicity of HctB leakiness in the discussion (lines 586-593) it is perhaps worth noting that HctA (ref 38) did not require a dual transcription/translation regulated system to obtain transformants. Is this due to less leakiness for the hctA riboswitch construct or differences in the impact of these proteins on development?

We elaborate on this in the discussion. Basically HctA does not appear to be as toxic even though there is some evidence of leakiness.

7) Lines 588-589: “Additionally, ectopic expression of HctB early in infection (16 hpi) inhibited the formation of infectious progeny.” Recommend replacing “inhibited” with significantly reduced. At least in my mind, inhibited implies a complete block in IFU production which is not the case (figure 6D).

We agree and have updated the sentence.

8) Recommend unifying the Y-axis formats in Fig_S1.

Agreed and fixed
