# Supplementary material for: Translational gene expression control in Chlamydia trachomatis
Source: PLoS One. 2022 Jan 27;17(1):e0257259. doi: 10.1371/journal.pone.0257259 (PMC8794103; doi:10.1371/journal.pone.0257259)
Supplement: Attachment — Submitted filename: rev_response.docx [file pone.0257259.s007.docx]

We have addressed the reviewers comments as follows:

Second review

Reviewer #2: The authors did an excellent job responding to the prior criticisms. Two minor points that should be addressed:

1) A file for Figure S2 (RNA-seq results) does not appear to be included with the revised submission. Page 35 is S1 and page 36 is S3. If the figure S2 intended for the revised submission matches the first submission, then I do not have additional comments for this data.

Yes this was an oversight on resubmission fig S2 is unchanged from the original submission and is now included in this submission

2) The authors indicate that they revised Figure S1 to unify the Y-axis formats in response to the round 1 reviewer comment. The included Figure S1 still uses 3 different numbering formats. Perhaps the incorrect version of the figure was uploaded. Recommend using scientific notation for panels A and B as shown in panels C and D.

We have fixed the graphs in fig S1 to all have the same style of Y axis

Best,

Scott Grieshaber
